# Cancer Treatment for Dual Eligibles: What Are the Costs and Who Pays?

**Published:** 2013-04-29

**Authors:** Susan G. Haber, Florence K.L. Tangka, Lisa C. Richardson, Susan A. Sabatino, David Howard

**Affiliations:** 1 RTI International; 2 Centers for Disease Control and Prevention, Division of Cancer Prevention and Control; 3 Rollins School of Public Health, Emory University

**Keywords:** Cancer treatment costs, Medicare, Medicaid

## Abstract

This study quantifies treatment costs for melanoma and breast, cervical, colorectal, lung, and prostate cancer among patients with dual Medicare and Medicaid eligibility. The analyses use merged Medicare and Medicaid Analytic eXtract enrollment and claims data for dually eligible beneficiaries age>18 in Georgia, Illinois, Louisiana, and Maine in 2003 (n=892,001). We applied ordinary least squares regression analysis to estimate annual expenditures attributable to each cancer after controlling for beneficiaries’ age, race/ethnicity, sex, and comorbid conditions, and state fixed effects. Cancers and comorbid conditions were identified on the basis of diagnosis codes on insurance claims. The most prevalent cancers were prostate (38.4 per 1,000 men) and breast (30.7 per 1,000 women). Dual eligibles with the study cancers had higher rates of other chronic conditions such as hypertension and arthritis than other beneficiaries. Total Medicare and Medicaid expenditures for dual eligibles with the study cancers ranged from $30,328 for those with lung cancer to $17,011 for those with breast cancer, compared with $10,664 for beneficiaries without the cancers. However, only 9% to 30% of medical expenditures for dual eligibles with the study cancers were attributable to the cancer itself. In 2003, combined Medicare/Medicaid spending for dual eligibles attributable to the six cancers in the four study states exceeded $256 million ($314 million in 2012 dollars). Dual eligibles with these cancers also had high rates of other medical conditions. These comorbidities should be recognized, both in documenting cancer treatment costs and in developing programs and policies that promote timely cancer diagnosis and treatment.

## Introduction

Cancer is a leading cause of illness and death in the United States. In 2007, more than 1.4 million new cancer cases were diagnosed and almost 560,000 people died from cancer in the United States [1]. Annual U.S. expenditures for cancer treatment increased from $24.7 billion in 1987 to an average of $48.1 billion during 2001 to 2005 [2]. The burden of cancer care falls disproportionately on the Medicare program, which provides insurance to 15% of the U.S. population [3] but covers 34% of cancer treatment costs [2].

Although previous studies have estimated the cost to Medicare of treating all cancers [2] and individual types of cancer [4–7], none have looked specifically at cancer treatment costs for people with dual eligibility for Medicare and Medicaid. Among people with such dual eligibility, Medicare is the primary payer for acute care services. Depending on a person’s income and assets, Medicaid may cover Medicare premiums, Medicare deductibles and coinsurance, and Medicaid services not covered by Medicare, including long-term care and, until 2006, outpatient prescription drugs.

Dually eligible beneficiaries are a vulnerable population whose characteristics differ from those of other Medicare beneficiaries in a number of ways that may influence their health care utilization. More than 60% have incomes below the federal poverty level. In addition, they have a lower average education level and are more likely to be from a minority population, to live alone, and to be institutionalized [8]. Combined Medicare and Medicaid spending for dually eligible beneficiaries is nearly five times higher than Medicare spending for Medicare beneficiaries not eligible for Medicaid, and Medicare spending alone is nearly twice as high [9]. Although dually eligible beneficiaries make up only 15% of the Medicaid population, they account for 39% of Medicaid spending [10]. Dually eligible beneficiaries also face the unique challenge of having to negotiate both the Medicare system and the Medicaid system. The establishment on December 30, 2010, of the Federal Coordinated Health Care Office, also known as the Medicare-Medicaid Coordination Office, within the Centers for Medicare & Medicaid Services (CMS) reflects heightened attention to the needs of dually eligible beneficiaries.

Although no previous research has been conducted concerning cancer treatment costs for dually eligible benficiaries, differences between dually eligible beneficiaries and other Medicare beneficiaries in the type of cancer treatment received and the stage of cancer at diagnosis have been documented [11–16]. These differences are likely to affect cancer treatment cost.

In this study, we used data from four states to estimate treatment costs for six types of invasive cancers—melanoma and breast, cervical, colorectal, lung, and prostate cancer—among patients with dual Medicare and Medicaid eligibility. We estimated costs for each program as well as combined Medicare and Medicaid costs. Because the prevalence of multiple chronic conditions is relatively high among all Medicare beneficiaries [17, 18] and especially high among those who are dually eligible [17], including dually eligible beneficiaries with cancer [12], we used multivariate regression analysis to estimate costs attributable to each of the six types of cancer while controlling for comorbid conditions and other beneficiary characteristics that may have an impact on costs.

## Materials and methods

We analyzed merged 2003 Medicare and Medicaid Analytic eXtract file (MAX) enrollment and claims data for dually eligible beneficiaries in four states: Georgia, Illinois, Louisiana, and Maine. MAX is a uniform dataset containing Medicaid eligibility, utilization, and payment information that CMS creates from Medicaid Statistical Information System (MSIS) data submitted by all U.S. states. We analyzed 2003 data because it was the most recent year for which MAX data were available when our study began.

We selected the four study states because fewer than 10% of their Medicaid beneficiaries were enrolled in capitated managed care plans in 2003. Although states are required to report encounter data for utilization by enrollees in capitated managed care plans, CMS does not recommend using encounter data for statistical analyses because reporting is incomplete and the accuracy of the reported encounter data is not validated [19]. The states also had high-quality cancer registry data, which were used in a companion study.

Because Medicare is the primary payer for most services for dually eligible beneficiaries, Medicare service coverage does not vary by state. However, Medicaid coverage for dually eligible beneficiaries in the four study states did vary. Illinois and Maine provided full Medicaid benefits for dually eligible beneficiaries with incomes up to 100% of the federal poverty level, whereas Georgia and Louisiana provided full benefits only for beneficiaries with incomes up to 75%. Although there may be state-level differences in provision of Medicaid services not covered by Medicare, we limited our analyses to services that were covered by Medicare and excluded the two main categories of Medicaid services not covered by Medicare during the study period (long-term care and outpatient prescription drugs).

We pooled data from the four states in order to have adequate numbers of dually eligible beneficiaries with cancer for our analysis. Because Medicare covers 91% of inpatient expenditures and 88% of ambulatory expenditures for dually eligible beneficiaries [9], we did not expect differences in state Medicaid policies to have an important effect on expenditures. Nevertheless, to control for variation in Medicaid eligibility and reimbursement policies, medical practice patterns, and provider supply, we included state fixed effects in our regression models.

The study population consisted of all residents of study states aged 18 or older who were dually eligible for Medicare and Medicaid and had at least 1 month of fee-for-service enrollment during 2003. Eligibility and utilization data for periods of enrollment in capitated managed care were excluded from the analyses. We included beneficiaries with less than 12 months of enrollment because they were a significant portion of dually eligible beneficiaries in the study states and excluding them could have biased our estimates. However, we excluded from our analyses eligibility and utilization data for these beneficiaries for periods during which they were enrolled in capitated managed care.

We calculated mean Medicare and Medicaid expenditures in each study state for beneficiaries with each of the six study cancers (melanoma and breast, cervical, colorectal, lung, and prostate cancer) and for all other beneficiaries combined, including those with nonstudy cancers. We then used multivariate regression to estimate marginal expenditures attributable to each study cancer after controlling for beneficiaries’ sociodemographic characteristics and comorbidities. We classified a beneficiary as having one the six study cancers if an International Classification of Diseases, 9th Revision, Clinical Modification (ICD-9-CM) diagnosis code for that type of cancer was in any inpatient Medicare or Medicaid claim for that beneficiary or in at least two claims on different dates for any other type of service for that beneficiary. Our prevalence and per capita cost estimates for each type of cancer were based on data for all dually eligible beneficiaries identified as having that type of cancer, including those who may no longer be receiving active treatment for their cancer.

We used ordinary least squares multivariate regression to estimate per-capita Medicare and Medicaid expenditures attributable to each of the six study cancers. All analyses were conducted with SAS software, version 9.2 (SAS Institute Inc., Cary, NC). Although researchers often use nonlinear two-part models to model health care expenditures to account for the large percentage of people who do not use any services, as well as skewness in the distribution of expenditures among service users, ordinary least squares has been used in some previous analyses of annual medical costs [20–22], particularly in analyses that, like ours, involved a very large number of study subjects.

We calculated the attributable cost of each type of cancer by subtracting predicted expenditures for beneficiaries without cancer from predicted expenditures for beneficiaries with the type of cancer in question while holding all other variables in the regressions constant at their mean level. Unlike other commonly used methods to estimate disease costs, the regression approach does not use diagnosis or procedure codes to determine whether specific claims are for care related to the disease in question. The regression approach also minimizes the extent to which the same medical expenditures are attributed to more than one disease by controlling for other diseases among people with multiple diseases [23, 24].

The regression models included variables indicating whether the individual had each of the six study cancers. In addition, the regressions controlled for beneficiaries’ age (expressed as a continuous variable), age squared (to account for non-linear effects of age), sex (male, with female omitted), race/ethnicity (black, Hispanic, and other, with white omitted), the presence of 25 diseases or medical conditions (including nonstudy cancers), and state fixed effects. We derived beneficiaries’ age, sex, and race/ethnicity from the Medicare denominator file and used procedures similar to those for determining their cancer status to determine whether they had each of the 25 categories of comorbid diseases or conditions (nonmelanoma skin cancers, other nonstudied cancers, carcinoma *in situ*, diabetes, hypertension, congestive heart failure, stroke, coronary heart disease, other cardiovascular diseases, asthma, back problems, chronic obstructive pulmonary disease, dyslipidemia, HIV/AIDS, injuries, pneumonia, pregnancy, renal failure, skin problems, arthritis, depression, organic psychoses, other mental health or substance abuse disorders, mental retardation, and degenerative diseases).

Depending on the regression model, the dependent variable was total per-capita expenditures for all types of services except long-term care and outpatient prescription drugs by Medicare, by Medicaid, or by both programs combined in 2003. As previously mentioned, we did not include expenditures for long-term care and outpatient prescription drugs because we were interested in the distribution of expenditures for services covered by both programs, and Medicare provides limited coverage for long-term care services and did not begin covering outpatient prescription drugs until 2006. Unadjusted mean per capita expenditures for long-term care services and outpatient prescription drugs did not differ significantly between dually eligible beneficiaries with and without cancer, suggesting that these services are not important contributors to cancer-attributable costs.

## Results

The final study population consisted of 892,001 dually eligible beneficiaries. Table 1 shows the age, race/ethnicity, sex, and state distribution of the study population in the four states by whether beneficiaries were diagnosed with any of the six study cancers. The comparison group includes both individuals without cancer and those with nonstudy cancers. However, less than 3% of beneficiaries in the comparison group had an invasive nonstudy cancer (Table 2). Beneficiaries with the six study cancers accounted for 62% of all beneficiaries with cancer in the study population (data not shown). Compared with beneficiaries in the comparison group, those with study cancers were older (mean age 75.1 years vs. 67.8 years), more likely to be white (67% vs. 64%), more likely to be male (38% vs. 34%), and less likely to have been enrolled for a full year (64% vs. 74%) (Table 1). Nearly half of all dually eligible beneficiaries in the study resided in Illinois, reflecting the larger size of that state’s population compared with the other study states.

Large percentages of dually eligible beneficiaries had the comorbid medical conditions included in the regression model (Table 2). For example, 59% had hypertension, 38% had arthritis, and 26% had diabetes. With the exception of HIV, pregnancy, and mental retardation, beneficiaries with one of the six study cancers were significantly more likely than other beneficiaries to have had each condition included in the model. For example, they were more than twice as likely to have pneumonia, almost twice as likely to have chronic obstructive pulmonary disease, and about 60% more likely to have congestive heart failure, coronary heart disease, and other cardiovascular diseases.

Among all dually eligible beneficiaries, prevalence rates (per 1,000 dually eligible beneficiaries of both sexes) were 20.2 for breast cancer, 13.0 for prostate cancer, 11.6 for colorectal cancer, 9.9 for lung cancer, 1.5 for cervical cancer, and 1.1 for melanoma (Table 3). The high overall prevalence of breast cancer, however, in part reflects the high percentage of women in the study population. Prostate cancer rates among men (38.4 per 1,000) were actually higher than breast cancer rates among women (30.7 per 1,000).

Combined Medicare and Medicaid annual per capita expenditures were substantially higher for dually eligible beneficiaries with the study cancers than for other dually eligible beneficiaries (Table 4). Mean combined expenditures were $30,328 for those with lung cancer, $27,418 for those with cervical cancer, $24,885 for those with colorectal cancer, and from about $17,000 to $19,000 for those with breast cancer, prostate cancer, and melanoma. In contrast, the average annual per capita expenditure for those who did not have any of the study cancers was $10,664. Although Medicare covered most expenses for all dually eligible beneficiaries, it covered 83–87% of costs for those with the study cancers compared with only 74% of costs for other dually eligible beneficiaries.

Although dually eligible beneficiaries with the study cancers had substantially higher per capita expenditures than other dually eligible beneficiaries, only a small portion of their annual expenditures was attributable to cancer (Table 5). Regression-adjusted annual per capita expenditures attributable to cancer ranged from approximately $9,000 for lung cancer to less than $1,700 for melanoma, and the share of per capita expenditures attributable to cancer ranged from 30% for beneficiaries with lung cancer to 9% for those with melanoma. The portion of these cancer-attributable costs paid by Medicare ranged from 89% for breast cancer to 95% for melanoma and prostate cancer. Expenditures attributable to cancer accounted for a substantially larger portion of mean per capita Medicare expenditures than mean per capita Medicaid expenditures. For example, they accounted for 32% of mean Medicare expenditures for dually eligible beneficiaries with lung cancer but only 13% of mean Medicaid expenditures for these beneficiaries.

## Discussion

In 2003, combined Medicare and Medicaid spending for dually eligible beneficiaries in the four study states attributable to the six study cancers was about $256 million, or 23% of total spending for these beneficiaries and 3% of spending for all dually eligible beneficiaries (excluding expenditures for prescription drugs and long-term care). In 2012 dollars, Medicare and Medicaid spending for the six cancers was about $314 million. Only 9% to 30% of medical expenditures for dually eligible beneficiaries with one the six types of cancers were attributable to the cancer itself. The relatively low proportion of expenditures attributable to the study cancers reflects the poor health status and high prevalence of comorbidities among dually eligible beneficiaries overall and especially among those with cancer. Dually eligible beneficiaries incurred substantial medical care costs for these comorbid conditions independent of their cancer status.

In addition, the study population included individuals in varying phases of cancer care, ranging from initial treatment to continuing care to terminal care. Although we were not able to control for phase of care in these claims-based analyses, results from previous studies have shown that cancer costs follow a U-shaped curve, with the highest costs near diagnosis and death [4, 7]. These findings suggest that the share of medical expenditures attributable to cancer would likely be higher for those patients receiving active treatment or end-of-life care.

Attributable per capita costs were highest for lung cancer and colorectal cancer patients, and lowest for melanoma and prostate cancer patients. These variations in per capita costs may be due to differences in factors such as treatment phase-specific costs and average duration of patient survival [7]. Study results have shown that inpatient services constitute a greater proportion of total adjusted long-term costs for colon and lung cancer patients than for breast or prostate cancer patients [25]. In addition, patients with lung or colorectal cancer have been found to survive for a shorter time after diagnosis than those with other cancers in our study [26]. These findings suggest that patients in our study with these two types of cancer were more likely to be receiving active treatment or end-of-life care, which have been shown to be the two most costly phases of cancer care [4, 25, 27]. Results from a study of Medicare beneficiaries in the initial phase of treatment showed that treatment costs for those with lung or colorectal cancer were about twice the costs for those with breast or prostate cancer [6]. Although these findings were consistent with ours, the level of expenditures was much higher, most likely because of the relatively high costs during the initial phase of treatment.

Our finding that Medicare covered most costs for dually eligible beneficiaries with cancer reflects Medicare’s role as the primary payer for most services other than long-term care services, for which Medicare provides limited coverage, and outpatient prescription drugs, which were not covered by Medicare during our study period. Although we excluded these services from our analyses, they were unlikely to have contributed significantly to cancer-attributable costs because per capita expenditures for these services differed little by beneficiaries’ cancer status.

This study had four notable limitations. First, because our cost estimates were based on data from 2003, they were less than what Medicare and Medicaid costs would be today. Taking into account inflation, costs in 2012 would be 25 percent higher than our 2003 cost estimates. Although these inflation-adjusted expenditure estimates do not account for increases in the number of cancer treatments or cost increases for cancer treatments that differ from general inflation [28], cancer spending as a percent of overall medical expenditures and expenditures by payer has remained constant over the past two decades [2]. This suggests that our estimates of the per capita burden of cancer expenditures for dually eligible beneficiaries to the Medicare and Medicaid programs are not seriously biased by using older data. However, our estimate of total expenditures attributable to the six cancers does not take into account changes since 2003 in the size of the dually eligible population, for example due to economic downturn or growth in the population over age 65. Second, our estimates of cancer-attributable costs were based on expenditures during the calendar year, not total treatment costs per diagnosed case. Third, these estimates include costs for all cancer patients (both incident and prevalent cases) and thus are lower than what the average annual cancer-attributable costs would have been for a patient in active treatment. We were not able to identify treatment phase because claims data do not report date of diagnosis. Claims data also do not specify cancer stage; thus, we were unable to estimate treatment costs by stage of disease. Fourth, because our analyses were based on data from only four states, our results may not be representative of costs for dually eligible beneficiaries in other states.

Unlike many other cost of illness studies, ours did not rely on reporting of specific procedure or diagnosis codes to identify cancer treatments. Instead, we used regression adjustment to compare all expenditures for dually eligible beneficiaries with the six study cancers with all expenditures for other dually eligible beneficiaries, while controlling for beneficiaries’ sociodemographic characteristics and comorbidities. We used individual disease indicators, rather than other commonly used methods of controlling for comorbidities, such as a comorbidity index, because our approach more accurately adjusts for an individual’s specific disease profile. A single comorbidity index variable is useful in analyses based on limited data for which a parsimonious model is important. However, parsimony was not a significant consideration for these analyses because of the large number of observations. Although our estimates were not affected by inaccuracies in reporting procedure and diagnosis codes to identify claims for cancer-related expenditures, they may have been affected by cancer-related bias in the identification of comorbid conditions. Comorbid conditions may be more likely to be diagnosed in people with cancer because cancer patients see a doctor more often than people without cancer. Such bias in the diagnosis of these conditions would have led to an underestimate of cancer-attributable costs.

Because of the availability of the Surveillance, Epidemiology, and End Results (SEER)-Medicare database, many analyses of cancer costs are based exclusively on direct Medicare reimbursements and do not reflect costs borne by secondary payers or by patients. Our findings indicate that the results of such analyses understate cancer costs among dually eligible beneficiaries by 5–11% depending on the type of cancer.

Although cancer-attributable expenditures for dually eligible beneficiaries with any of the six types of cancers included in this study were considerable, most health care expenditures in this population were not attributable to cancer itself but rather to other serious medical conditions. Although dually eligible beneficiaries in general tend to have a high prevalence of these conditions, we found the prevalence rates to be even higher among dually eligible beneficiaries with cancer, and these other conditions accounted for a large proportion of their health care expenditures.

These findings, which highlight the complexity of service needs for dually eligible beneficiaries with cancer and the joint role of Medicare and Medicaid in covering these services, provide valuable new information that can help inform initiatives, such as those from the new Federal Coordinated Health Care Office, designed to ensure that dually eligible beneficiaries have access to seamless, high-quality, cost-effective health care. Dually eligible beneficiaries often must see multiple providers as a result of their substantial health problems, and a cancer diagnosis only increases the complexity of their service needs and the challenges they face in accessing necessary services. These challenges may be a factor in previously documented disparities between dually eligible beneficiaries and other Medicare beneficiaries in the stage of their cancer at diagnosis and in the type of cancer treatment they receive [11–14]. The challenges in navigating the health care system that are encountered by anyone with multiple health problems are heightened for dually eligible beneficiaries, who are poorer and often less educated than other Medicare beneficiaries and who must simultaneously negotiate the Medicare and Medicaid programs. The impact of comorbidities on dually eligible beneficiaries with cancer should be recognized, not only in documenting cancer treatment costs, but also in developing programs and policies that promote timely cancer diagnosis and treatment.

## Figures and Tables

**Table 1:** Distribution of Characteristics Among Dually Eligible Beneficiaries Aged 18 or Older

	Beneficiaries with a Study Cancer^1^	All Others^2^		All
Age (mean)	75.1	67.8	***	68.2
Race/ethnicity (%)			***	
White	67.3	64.2		64.4
Black	29.6	31.1		31.0
Hispanic	1.0	1.7		1.6
Other	2.1	3.1		3.0
Sex (%)			***	
Female	62.2	66.2		66.0
Male	37.8	33.8		34.0
State (%)			***	
Georgia	26.2	27.8		27.7
Illinois	50.6	47.7		47.8
Louisiana	16.8	18.0		18.0
Maine	6.4	6.5		6.5
Enrolled for 12 months (%)	64.4	74.4	***	73.9
N	48,809	843,192		892,001

*** Significantly different from beneficiaries with a study cancer at *p* <.001

1 includes beneficiaries with any of the six study cancers (melanoma or breast, cervical, colorectal, lung, or prostate cancer).

2 Includes beneficiaries with cancers other than the six study cancers.

**Table 2:** Percentage of Dually Eligible Beneficiaries Aged 18 or Older with Selected Comorbid Conditions

Comorbid Condition	Beneficiaries with a Study Cancer^1^	All Others^2^		All
	%	%		%
Diabetes	30.5	25.6	***	25.9
Hypertension	71.0	57.8	***	58.5
Congestive Heart Failure	22.0	13.8	***	14.2
Stroke	11.8	8.6	***	8.8
Coronary Heart Disease	27.1	16.7	***	17.3
Other Cardiovascular Diseases	49.5	30.9	***	32.0
Asthma	7.0	5.3	***	5.4
Back Problems	15.5	12.0	***	12.2
Chronic Obstructive Pulmonary Disease	27.9	14.1	***	14.8
Depression	11.9	11.5	*	11.5
Dyslipidemia	20.9	17.4	***	17.6
HIV	0.2	0.7	***	0.7
Injuries	29.5	20.6	***	21.1
Pneumonia	16.0	7.3	***	7.7
Pregnancy	0.2	0.4	***	0.3
Renal Failure	9.0	6.3	***	6.5
Skin Problems	20.1	15.4	***	15.6
Arthritis	49.1	37.2	***	37.9
Mental Health/Substance Abuse	21.0	17.7	***	17.8
Organic Psychoses	11.8	8.7	***	8.9
Mental Retardation	1.1	3.0	***	2.9
Degenerative Diseases	9.1	7.3	***	7.4
Cancer *In Situ*	8.2	0.6	***	1.1
Nonmelanoma Skin Cancer	2.3	1.0	***	1.0
Other Nonstudy Cancers	11.1	2.6	***	3.0
N	48,809	843,192		892,001

*** Significantly different from beneficiaries with a study cancer at *p* <.001

* Significantly different from beneficiaries with a study cancer at *p* <.05

1 Includes beneficiaries any of the six study cancers (melanoma or breast, cervical, colorectal, lung, or prostate cancer).

2 Includes beneficiaries with cancers other than the six study cancers.

**Table 3:** Prevalence of Study Cancers per 1,000 Dually Eligible Beneficiaries Aged 18 or Older

Cancer Type	Overall Prevalence	Gender-specific Prevalence
Breast cancer	20.2	30.7 (women only)
Cervical cancer	1.5	2.3 (women only)
Colorectal cancer	11.6	N/A
Lung cancer	9.9	N/A
Melanoma	1.1	N/A
Prostate cancer	13.0	38.4 (men only)

N/A, not applicable (cancer is not gender specific)

**Table 4:** Mean Annual Per Capita Expenditures for Dually Eligible Beneficiaries Aged 18 or Older

	Total Expenditures		Medicare Expenditures			Medicaid Expenditures		
	
Cancer Type	Mean $	Standard Deviation $	Mean $	Standard Deviation $	Percent of Total	Mean $	Standard Deviation $	Percent of Total
Breast	17,011	22,430	14,144	19,550	83.1	2,867	7,768	16.9
Cervical	27,418	31,099	23,130	27,789	84.4	4,288	9,466	15.6
Colorectal	24,885	28,060	21,534	24,760	86.5	3,351	10,230	13.5
Lung	30,328	28,138	26,430	25,535	87.1	3,897	8,601	12.8
Melanoma	18,737	22,736	15,528	18,922	82.9	3,208	10,635	17.1
Prostate	18,131	22,154	15,608	19,947	86.1	2,523	6,779	13.9
All others^1^	10,664	21,472	7,900	17,111	74.1	2,764	10,581	25.9

1 Includes beneficiaries with cancers other than the six study cancers

**Table 5:** Annual Per Capita Expenditures Attributable to Cancer for Dually Eligible Beneficiaries Aged 18 or Older

	Total Expenditures		Medicare Expenditure				Medicaid Expenditures					
	
Cancer Type	Attributable Cost $	Standard Error $		Percentage of Mean Expenditures	Attributable Cost $	Standard Error $		Percentage of Mean Expenditures	Attributable Cost $	Standard Error $		Percentage of Mean Expenditures
Breast	3,191	128	***	18.8	2,854	100	***	20.2	336	74	***	11.7
Cervical	6,269	459	***	22.9	5,838	357	***	25.2	431	265		10.0
Colorectal	7,325	168	***	29.4	6,864	131	***	31.9	461	97	***	13.8
Lung	9,015	185	***	29.7	8,502	143	***	32.2	513	107	***	13.2
Melanoma	1,641	553	**	8.8	1,564	430	**	10.1	77	319		2.4
Prostate	2,834	161	***	15.6	2,704	125	***	17.3	130	93		5.1

*** Significantly different from 0 at *p* <.001

** Significantly different from 0 at *p* <.01
